# Effect of centerline marking on lane changing behavior of drivers

**Published:** 2019-08

**Authors:** Jayalath Edirisinghe, Vasantha Wickramasinghe

**Affiliations:** ^*a*^Senior lecturer at University of Peradeniya, Sri Lanka.; ^*b*^University of Peradeniya, Sri Lanka.

## Abstract

**Background:**

Traffic accidents are in continuous rise on Sri Lankan roads. According to the records available at traffic headquarters, one death is reported at every 3 hour or less on Sri Lankan roads. Similar to the rest of the world, driver misbehavior contribute heavily on those road mishaps among identified reasons for common road traffic accidents in Sri Lanka, careless overtaking has recorded as the number one reason. Therefore, this paper is about a study carried out to study the effect of centerline markings on lane changing behavior of drivers.

**Methods:**

Three road segments from the Sri Lankan main roads were selected for this study. All the roads were single carriageways with one lane per direction. Out of three road segments, two were mountainous and winding and one is flat and rolling.

For each road segment, non-lane changeable segments were divided into three types. They were Length less than 100 m (L1), Length between 100 m and 300 m (L2) and length greater than 300 m (L3). Vehicle movement behavior were observed using video cameras and number of overtaking were collected after the field surveys of one hour each for each kind of non- changeable lane segments. Video cameras were installed to observe vehicle movement at non- lane changeable as well as legally acceptable lane changes. With respect to non- lane changeable segments, both the beginning of non-lane changeable areas as well as the ending of non – lane changeable segments were observed.

**Results:**

Following tables are the summary of observations made based on video recordings.

The next table is the summary of overtaking observed at the beginning and the end of non- lane changeable segments;

According to the field measurements, it was clear that there is a high tendency for drivers to overtake when the continuous length of non-changeable road segments (single line or double line divided) is longer than 300 m. Further, chances were slightly higher when there is a bunching. Bunching is due to slow moving vehicle controlling the optimum speed of most of the other vehicles in the bunch.

**Conclusions:**

From the summarized results of this study, it is evident that drivers are forced to change the lane to overtake vehicle in front of them when the length of the non- lane changeable lane length is longer. More frequent when there is a bunching effect due to slow moving vehicles.

Further, lane changing to overtake vehicles is more prominent towards the latter part of the non-lane changeable section than the beginning. It may be an indication that all the drivers are not really indiscipline in changing the lane and overtaking but they may be frustrated after driving a long distance without having opportunity to go at their comfortable speed.

Therefore, introducing the overtaking lanes at regular intervals or any other such suitable measures can be an answer to such illegal lane changing to overtaking. Though there are no adequate details to prove, such overtaking may lead to a traffic accident.

**Keywords:**

Road marking, Driver behavior

